# Hyperbaric Oxygen Therapy and Mild Hyperbaric Oxygen Therapy Are Not Synonymous: A Narrative Review

**DOI:** 10.3390/medsci14030360

**Published:** 2026-06-30

**Authors:** Mark Morningstar, Megan Strauchman

**Affiliations:** Department of Hyperbaric Medicine, Natural Wellness and Pain Relief Center, Grand Blanc, MI 48439, USA; strawk_man@yahoo.com

**Keywords:** hyperbaric oxygenation, oxygen inhalation therapy, hyperoxia, reactive oxygen species

## Abstract

**Background/Objectives**: Hyperbaric oxygen therapy (HBOT) and mild hyperbaric oxygen therapy (mHBOT) are two terms commonly used by hyperbaric chamber sellers, hyperbaric oxygen therapy practitioners, and even by many scientists as if they were synonymous. The following review of hyperbaric oxygen therapy goes into the relevant aspects that relate to the clinical uses of mHBOT and HBOT, such as pressure, the percentage of oxygen in inspired gas, the amount of oxygen dissolved in plasma, and biological effects, as they apply to a broad range of diseases that can be treated with mHBOT and HBOT. **Methods**: PubMed was searched from January 1990 to March 2026 using combinations of the following example terms: “hyperbaric oxygen therapy,” “hyperbaric oxygen,” “mild hyperbaric oxygen,” and “mild hyperbaric therapy.” Additional articles were identified through a reference list review and consensus documents from UHMS, ECHM, EUBS, and ANZHMG. Articles were selected based on relevance to pressure classification, oxygen delivery parameters, physiological mechanisms, clinical outcomes, and terminology standardization. **Results**: The use of inconsistent terminology for hyperbaric systems has become a serious problem, particularly because low-pressure soft-chamber systems are called “HBOT” but have very different mechanisms of action and even different regulatory requirements. **Conclusions**: HBOT and mHBOT are not equivalent medical interventions. A set of classification criteria for the proper characterization of pressure, oxygen, and chamber should be formulated. The issues of terminology overlap should be addressed, and how this overlap affects our perception of research data, patients’ expectations, clinician–church communication, reimbursement issues, and scientific criteria for experimental design will be discussed.

## 1. Introduction

Hyperbaric oxygen therapy (HBOT) is the administration of 100% oxygen under compressed gas in a hyperbaric chamber. To solubilize in plasma at extreme amounts, additional oxygen is transported to tissues that require oxygen at elevated pressures and concentrations. HBOT has many effects on biological systems. Angiogenesis, or the formation of new vessels, the release of stem cells, anti-inflammatory effects, and even anti-microbial effects are but a few of the many effects that HBOT has on the body. For decades, HBOT has been used in various clinical applications. HBOT has long been a mainstream medical treatment with extensive scientific research and numerous controlled clinical studies [1,2]. In fact, there are many indications for which HBOT is an established treatment, with well-designed documentation of its use in treating human disease [3,4].

Another commonly used term in both clinical and commercial settings is mild hyperbaric oxygen therapy (mHBOT). This intervention is typically conducted in a low-pressure soft chamber. Typically, this intervention is conducted using ambient air (21% oxygen) or oxygen generated from an oxygen concentrator. Increased partial pressure of oxygen in the tissues of patients receiving mHBOT is not of sufficient magnitude or duration to elicit most of the effects described for HBOT. As this review will outline, the term mHBOT should be used in clinics that use a soft chamber for various wellness applications and to aid recovery. The biological effects of mHBOT are distinct from those reported for HBOT.

The terms “hyperbaric oxygen therapy” and “HBOT” are used to describe the treatment given in a hard chamber by contrast. While these terms are often used correctly in clinical studies, there is considerable confusion in the treatment of various conditions in the commercial market and in the research literature. Confusion arises when evidence from studies conducted at high pressures is inappropriately used to support claims about the effects of mHBOT. Evidence supporting HBOT should not automatically be assumed to apply to mHBOT because the pressure, oxygen dose, and resulting physiological responses may differ substantially between interventions. The dose of oxygen delivered to tissues during high-pressure treatment is not delivered during mHBOT. Several authors in the field of hyperbaric medicine have recently addressed this problem and provided a clear definition of HBOT that differentiates medical HBOT from other interventions [3,5,6].

There is growing concern in the medical community as terminology associated with HBOT is being inappropriately extended to support evidence for lower-pressure interventions. In numerous instances, low-pressure or mild hyperbaric chamber systems may be used to treat various medical problems for which there is little or no evidence to support their use. Such misunderstandings, which have potential for patient harm and/or public deception, arise from two main sources: (1) extension of dose-responsive evidence of HBOT to pressures that do not produce equivalent oxygen effects, and (2) unclear definitions and delivery parameters of interventions referred to using HBOT terminology.

At higher pressures, the specific mechanisms of action for HBOT treatment of various diseases and conditions are activated. These include increased angiogenesis, stem cell mobilization, reduced inflammatory responses, and antibacterial effects. However, at lower pressures, these same mechanisms may not be activated to a clinically meaningful degree. Thus, while high-pressure HBOT has a solid foundation of high-quality studies (primarily randomized controlled studies and consensus documents), evidence for mHBOT is very limited, of poor methodological quality, and often incorrectly extrapolated from studies of HBOT.

The primary purpose of this review is to examine whether hyperbaric interventions delivered across substantially different pressure and oxygen ranges should be considered equivalent therapies within scientific literature, clinical communication, and research design. A secondary objective is to propose a classification framework that distinguishes conventional HBOT from lower-pressure hyperbaric exposures while recognizing that both may possess biological activity. Importantly, the purpose of this review is not to suggest that lower-pressure hyperbaric exposures are biologically inactive.

## 2. Methods and Materials

This narrative review was conducted to examine the distinctions between conventional hyperbaric oxygen therapy (HBOT) and lower-pressure hyperbaric exposures, frequently described as mild hyperbaric oxygen therapy (mHBOT). Literature searches were performed in PubMed, Scopus, and Web of Science between January 2026 and March 2026. Search terms included (1) hyperbaric oxygen therapy; (2) hyperbaric oxygen; (3) mild hyperbaric oxygen; (4) mild hyperbaric oxygen therapy; and (5) hyperbaric medicine. Boolean combinations of these terms were utilized where appropriate. Articles published between 1990 and March 2026 were considered. Studies were selected based on relevance to hyperbaric pressure classification, oxygen dose–response relationships, molecular and physiological mechanisms, clinical outcomes, terminology and nomenclature, and regulatory and consensus definitions. Preference was given to randomized controlled trials, systematic reviews, consensus statements, society position papers, and mechanistic studies relevant to the biology of hyperbaric oxygen.

## 3. Hyperbaric Oxygen Therapy Pressure Standards and Definitions

HBOT is typically delivered in a hard chamber, where 100% oxygen is delivered at pressures above normal atmospheric (ambient) pressure. Hyperbaric oxygen refers to any pressure greater than 1.0 ATA, but, in medical hyperbaric oxygen therapy, a minimum pressure is typically defined to delineate treatment.

The minimum pressure required for HBOT is between 1.4 ATA and 1.5 ATA for clinical applications worldwide. Given the variety of conditions for which HBOT is used as a treatment, the typical pressure for these hospital-based treatments is greater than 2.0 ATA at 100% oxygen. In the US, the Undersea and Hyperbaric Medical Society (UHMS) defines clinical HBOT as a treatment at greater than 1.4 ATA with 100% oxygen [1]. Despite this minimum, nearly all FDA-cleared indications, including decompression illness, carbon monoxide poisoning, radiation injury, necrotizing infections, and ischemic wounds, are treated at pressures between 2.0 and 3.0 ATA [1,2]. The UHMS further states that interventions delivered below 1.4 ATA do not constitute HBOT for medical conditions, with the sole exception of treatment for acute mountain sickness [1].

The European Committee for Hyperbaric Medicine (ECHM) of the European Federation of Hyperbaric Associations (EF Hib Ass) defines hyperbaric oxygen therapy (HBOT) as the administration of oxygen at increased pressure above ambient. For 100% oxygen to be therapeutic, the partial pressure of oxygen in the inspired gas must exceed 1.5 ATA [3,6]. Thus, the chamber absolute pressure must be raised above 1.5 ATA. Typical treatment pressures for the effective management of several defined medical conditions are set at 2.0 to 2.8 ATA, in accordance with guidelines established by the Undersea and Hyperbaric Medical Society (UHMS). Position statements regarding “soft-sided” or low-pressure HBOT (treatment delivered at less than 1.5 ATA) have been published by ECHM and the European Underwater and Baromedical Society (EUBS) [6]. These statements point to a lack of high-quality studies supporting a medical indication for the use of such systems in hyperbaric oxygen therapy.

The Australian and New Zealand Hyperbaric Medicine Group (ANZHMG) defines HBOT as the administration of 100% oxygen at a pressure greater than atmospheric (1.0 ATA) [7]. In practice, however, treatments are commonly delivered at pressures greater than 1.5 ATA. As per established protocols in Australia and New Zealand, typical treatment pressures for HBOT range from 2.0 to 3.0 ATA [7].

The standards for hyperbaric oxygen therapy around the world define HBOT as delivered at less than 1.5 ATA, and therefore outside the evidence base for all indications for which HBOT is used. Pressures delivered in the range of 1.2 to 1.49 ATA have been described as “mild hyperbaric” or “wellness pressurization” in HBOT commerce and are not indicated for any of the medical conditions for which there is evidence to support the use of HBOT. These treatments should be judged against the standards of hyperbaric medicine.

### Rationale for Classification Thresholds

The classification framework proposed in Table 1 was not developed from scratch but rather relies on already existing recommendations, position papers, and more from all relevant hyperbaric medicine organizations such as the Undersea and Hyperbaric Medical Society (UHMS), the European Committee for Hyperbaric Medicine (ECHM), the European Underwater and Baromedical Association (EUBS), and the Australasian Hyperbaric Association (ANZHMG). Around 1.4–1.5 ATA of near-100% oxygen breathed during conventional HBOT is thus approximated using current clinical practice guidelines for hyperbaric medicine.

**Figure 1:**
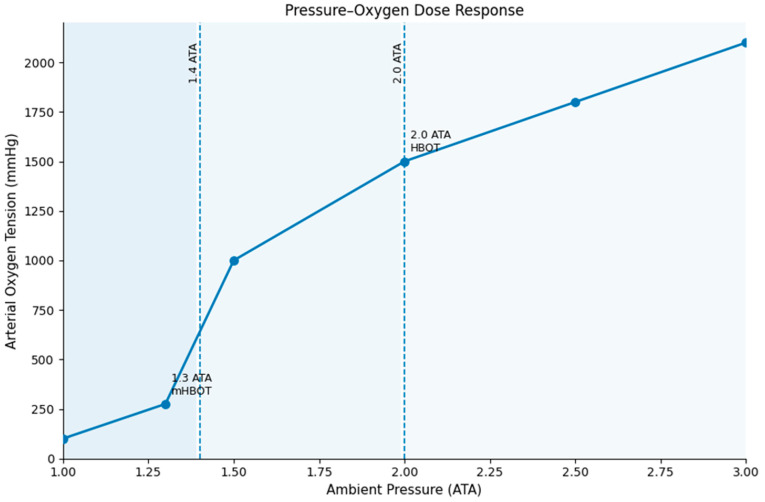
Arterial oxygen tension increases nonlinearly with rising ambient pressure, with disproportionately greater gains occurring above approximately 1.5 ATA when breathing high-concentration oxygen. Mild hyperbaric exposures (1.2–1.49 ATA) produce relatively modest increases in oxygen tension compared to medical-grade hyperbaric oxygen therapy (≥2.0 ATA). This divergence supports classifying these interventions as physiologically and clinically distinct modalities.

Figure 1 is not intended to represent a sharp, biological cut-off at 1.5 ATA. Instead, it establishes a threshold pressure, which is the lower limit for conventional hyperbaric treatment, as defined by the current clinical practice guidelines, definitions within hyperbaric medicine and related health care, and practices and regulations within the health care industry. This pressure is typically the lower limit of pressure used in studies of hyperbaric oxygen therapy and is used in most instances of conventional hyperbaric treatment, as recommended by position statements and recommendations from organizations involved in hyperbaric medicine, such as the UHMS, ECHM, EUBS, and ANZHMG.

Throughout the manuscript, the term 1.5 ATA will be used as a practical classification threshold. This is approximately the lower boundary of the threshold used by all major organizations involved in hyperbaric medicine, and it is around this value that the majority of HBOT clinical protocols have been developed. Some oxygen-responsive processes may commence below this value, but many others become more evident as oxygen tension increases beyond the pressures typically used for conventional HBOT. The value of 1.5 ATA is best used as a threshold for a clinical and/or regulatory tool and not as some critical and unique transition for particular biological processes.

## 4. “Soft-Sided” vs. “Hard-Sided” Hyperbaric Chambers

The term “soft-sided” in this context typically refers to mild hyperbaric oxygen chambers, typically made of durable PVC or nylon. These near-ambient-pressure systems typically operate between 1.1 and 1.4 ATA, using either ambient air or a high-oxygen mixture delivered from an oxygen concentrator (see Figure 1). Soft chambers are considered wellness devices and are typically not regulated in the same manner as medical devices. In general, soft-sided chambers are not limited to use under the supervision of a licensed healthcare provider; they are typically used for general wellness purposes and are not used to treat specific conditions. Treatment with soft-sided chambers is typically not covered by insurance because they are not used to treat medical conditions. Chambers made of soft materials are classified as Class II medical devices in the US if marketed for the treatment of mild conditions, but they are considered wellness devices or equipment and not medical devices when used for general wellness. As such, soft-sided chambers are not FDA approved for the treatment of any medical condition requiring increased pressure.

Hard-sided hyperbaric chambers typically consist of a tube or cylindrical compartment, approximately 7–10 feet in length and 24–40 inches in diameter, made of sturdy, durable steel or high-quality acrylic. They can reach pressures of 2.0–3.0 ATA or higher. These hard-sided hyperbaric chambers are operated in a 100% oxygen environment at elevated pressures. The hard-sided tube hyperbaric chamber is typically prescribed for patients suffering from severe medical conditions such as decompression sickness and for patients suffering from the effects of radiation. Many people with chronic non-healing wounds are also prescribed HBOT in a hard-sided hyperbaric chamber. Hard-sided hyperbaric chambers are used in a clinical or hyperbaric hospital setting. Hard-sided hyperbaric chambers are classified as either Class II or Class III medical devices and are regulated by the FDA. These powerful chambers are operated by a certified hyperbaric technician, who is supervised by a licensed medical doctor. Many insurance companies cover the cost of HBOT for patients who undergo treatment in hard-sided hyperbaric chambers for FDA-approved indications and under the supervision of a qualified medical doctor.

## 5. Misapplication of HBOT Evidence to Mild Hyperbaric Systems

Much of the peer-reviewed hyperbaric literature regarding its many potential therapeutic uses may be misapplied to mHBOT. Studies are generally misapplied in one of two primary ways: (1) the studies of HBOT are generally misapplied to mHBOT studies; (2) studies of mild hyperbaric exposures (i.e., “mild HBO” or mHBOT) are sometimes misapplied to justify the use of high-pressure treatment with 100% oxygen. The critical parameters (e.g., pressure, percentage of oxygen, treatment duration, and frequency) are often quite different for HBOT versus mHBOT studies, and it is important that each be studied independently and that data be applied accordingly. The scientific foundation of HBOT is built upon well-characterized dose–response relationships between ambient pressure, inspired oxygen concentration, and resulting tissue oxygen tensions. At treatment pressures typically ranging from 2.0 to 3.0 atmospheres absolute (ATA) with near-100% oxygen, HBOT produces substantial increases in dissolved plasma oxygen, often exceeding 1500–2000 mmHg arterial oxygen tension. These supraphysiologic oxygen levels drive mechanisms such as angiogenesis, stem cell mobilization, modulation of inflammatory pathways, and enhanced antimicrobial activity. Critically, many of these mechanisms are dependent on pressure and require oxygen partial pressures that are unattainable at lower ambient pressures.

In contrast, mHBOT systems operating at approximately 1.2 to 1.4 ATA and typically utilizing ambient air or an oxygen concentrator produce comparatively minimal increases in tissue oxygenation. The resulting oxygen partial pressures are substantially lower than those achieved in standard HBOT protocols and fall below thresholds associated with many of the therapeutic mechanisms cited in the HBOT literature. As such, the biological equivalence of these interventions cannot be assumed.

Despite these well-established physiological differences, there is increasing evidence that findings from high-pressure HBOT studies are routinely extrapolated to justify the use of mHBOT [8]. This extrapolation represents a fundamental category error: it applies outcomes observed under one set of treatment conditions, defined by significantly higher pressure and oxygen delivery, to a distinct intervention with materially different exposure parameters. In pharmacologic terms, this would be analogous to applying clinical trial data obtained at a full therapeutic dose to support the efficacy of a substantially lower, subtherapeutic dose without dose–response validation.

This misapplication is most apparent in the marketing and educational content of clinics utilizing low-pressure systems. In these contexts, providers frequently reference peer-reviewed studies involving HBOT delivered at ≥2.0 ATA while offering treatments at approximately 1.3 ATA. The cited outcomes, such as neurologic recovery, enhanced wound healing, or anti-inflammatory effects, are therefore attributed to an intervention that does not replicate the conditions under which those outcomes were demonstrated. Importantly, this practice is often not accompanied by clear disclosure of chamber pressure, oxygen concentration, or the distinction between HBOT and mHBOT, creating a substantial risk of patient misunderstanding.

The implications of this misrepresentation are both scientific and clinical. From a research standpoint, conflating HBOT and mHBOT undermines the integrity of the evidence base by obscuring the relationship between treatment dose and outcome. It complicates efforts to synthesize data across studies, contributes to heterogeneity in reported results, and may lead to erroneous conclusions regarding efficacy. From a clinical perspective, the use of HBOT terminology to describe mHBOT interventions may lead patients to expect outcomes consistent with medical-grade therapy, potentially delaying access to evidence-based treatments or diverting resources toward interventions with unproven benefit.

The absence of standardized terminology allows these terms to remain interchangeable in practice, facilitating the ongoing misapplication of evidence. This gap highlights the need for coordinated action among governing and scientific bodies, including the International Hyperbarics Association, UHMS, ECHM, and ANZHMG, to establish clear, globally recognized definitions.

Such classifications should, at a minimum, delineate therapies based on pressure thresholds, oxygen delivery parameters, and intended clinical use. Standardized terminology would not only improve scientific clarity but also enhance patient understanding, support appropriate clinical decision-making, and reduce the potential for misleading claims. Without these measures, the distinction between HBOT and mHBOT will remain blurred, and the misapplication of evidence is likely to persist.

In summary, the current landscape reflects a systematic conflation of two physiologically and clinically distinct interventions. The continued use of HBOT evidence to support mHBOT raises critical methodological and ethical concerns. Establishing a universal classification standard is therefore essential to preserving the integrity of hyperbaric medicine and ensuring that therapeutic claims are aligned with the underlying evidence base.

## 6. Pressure-Dependent Molecular Responses to Hyperoxia

Hyperbaric oxygen therapy (HBOT) is delivered in a chamber where ambient pressure and oxygen concentration are increased. The increase in ambient pressure and oxygen concentration is related, but the physiological and cellular effects of increased oxygen concentration are not directly related to the increase and do not follow a simple linear relationship. Many of the physiological and cellular effects are threshold dependent, and, as a result, the dose–response relationships are frequently nonlinear. For many signaling pathways, a moderate increase in oxygen concentration can stimulate adaptive responses, but, at higher oxygen concentrations, the same pathways can be triggered to produce less beneficial physiological effects. Understanding the molecular effects of increased pressure and oxygen exposure is crucial for determining whether mild HBOT (mHBOT) and conventional HBOT (cHBOT) should be considered equivalent interventions.

There is an increase in the concentration of dissolved oxygen in the plasma as a result of hyperbaric exposure. For treatments conducted at pressures between 2.0 and 3.0 ATA while breathing 100% O_2_, arterial oxygen tensions exceed 1500 mmHg. This is many times greater than for the administration of O_2_ at ambient pressure and much greater than for mHBOT. Thus, high concentrations of dissolved O_2_ in the blood can penetrate hypoxic tissues and stimulate several signaling pathways that use O_2_ as a substrate [4,9].

A well-studied class of molecules affected by hyperoxia is reactive oxygen species (ROS). Initially viewed solely with trepidation as highly damaging metabolic byproducts, ROS have recently been recognized as important signaling molecules that control a wide array of gene expression programs. Included in this list are programs controlling aspects of the cell involved in inflammation, angiogenesis, and the cell’s response to various other stresses. The three major families of transcription factors affected by hyperoxia and thereby modulating gene expression are the redox-sensitive Nrf2, NF-κB, and AP-1 families. The ROS produced by cells under hyperoxia stimulates a hormetic response; that is, while high levels of ROS can be very damaging to cells, low to moderate amounts of ROS can trigger various highly beneficial adaptive responses in cells [10,11,12].

Hypoxia-inducible factor-1α (HIF-1α) is another important molecular target of hyperoxia. HIF-1α-activated pathways for growth and repair have recently been the subject of several studies investigating the repeated intermittent hyperoxia effects of HBOT. The term hyperoxia-induced-hypoxia, or the hyperoxic–hypoxic paradox, has recently been proposed to explain the mechanisms of HBOT-induced angiogenesis, stem cell mobilization, and tissue repair. The fluctuations in oxygen levels during higher-pressure exposures may be sufficient to activate HIF-1α and induce the expression of vascular endothelial growth factor (VEGF) and other angiogenic growth factors, such as fibroblast growth factors (FGFs). In addition, other factors that are involved in stem cell mobilization, such as stromal-derived factor-1 (SDF-1), may also be induced [12,13].

The best-studied effect of HBOT is an increase in the expression of VEGF and other growth factors involved in angiogenesis. There is also an increase in the expression of other growth factors, including fibroblast growth factors (FGFs). In addition to increasing the expression of growth factors involved in angiogenesis, HBOT has been shown to increase the expression of endothelial nitric oxide synthase (eNOS), an enzyme involved in the production of nitric oxide, a potent vasodilator. Increased expression of growth factors involved in angiogenesis has been shown to contribute to wound healing, as well as to the treatment of radiation injury and other ischemic diseases. While it is possible that mild HBOT may have some effect on some of these growth factors, there is very limited information on the degree to which they are increased by mild HBOT. A comparison of the effects of mild HBOT and HBOT on these growth factors is not possible. The relationship between oxygen tension and the expression of growth factors involved in angiogenesis appears to be nonlinear: the expression of these growth factors may increase slowly with increasing oxygen tension up to a threshold and then increase dramatically [4,14,15,16].

Stem cell mobilization by HBOT also results in increased numbers of circulating stem cells, particularly CD34+ cells. It is thought that HBOT increases nitric oxide levels, which in turn increase the number of stem cells that migrate to sites of injury and participate in tissue repair. The use of HBOT has been reported to result in the complete healing of chronic wounds and in the treatment of individuals who have received radiation for cancer. There are limited data available on the effects of sub-1.5 ATA exposures on stem cell mobilization. More research is clearly warranted in this area [17,18].

Several studies report improved mitochondrial function in various models with HBOT. There is evidence that HBOT can increase the mitochondrial reserve capacity or enhance cellular energy production in various cell types. Such enhanced mitochondrial function may, in part, account for improved neuronal function following HBOT. In addition, improved wound healing and increased exercise performance in patients have been reported. The pressure required for such mitochondrial adaptive responses has not been established, and similar qualitative effects may occur at pressures below 1.5 ATA of oxygen and at a different magnitude [19,20].

The numerous pathways sensitive to hyperoxia are typically activated along a continuum or dose–response curve, with the threshold point usually unknown. It can thus only be assumed that, at some point during the increase in oxygen exposure, a critical level is required to induce activation of the relevant pathway(s). Consequently, just because data demonstrate that many biological effects are induced by HBOT at sub-1.5 ATA exposure, this does not mean that all of these results have clinical relevance. Activation of a pathway, changes in gene and protein expression, and increases in biomarkers and various physiological processes do not necessarily translate into therapeutic benefit for specific clinical conditions.

Stimulation of a molecular pathway in experiments on physiological effects does not automatically translate to clinical efficacy. The experimental evidence of mechanisms of action derived only from experiments on physiologic effects is insufficient to clarify the differences between HBOT and mHBOT. Evidence of therapeutic effects is provided only by well-controlled clinical trials. The differences between HBOT and mHBOT must therefore be proven by the sum of the effects on the organism, the effects on molecules, and the evidence of clinical efficacy.

The clinical significance of these enhanced processes (i.e., angiogenesis, stem cell mobilization, and modulated inflammation) must be demonstrated by assessing the extent to which they can be increased by low-pressure treatments to induce clinically relevant effects comparable to those described for conventional HBOT. While enhanced angiogenesis will most likely support wound healing and the recovery of irradiated and chronically ischemic tissues by enabling them to become better oxygenated, the extent to which such effects can be induced by low-pressure treatments and still meet the threshold for clinically relevant therapeutic applications remains to be established. This, however, can only be addressed by future studies, which will have to correlate the molecular effects of treatments with the health status of treated patients.

## 7. Peer-Reviewed Literature Using Sub-1.5 ATA Exposures

There is a large body of peer-reviewed literature describing sub-1.5 ATA exposures, typically using ambient air and/or adding oxygen to it to create mixtures with moderate oxygen concentrations. Most studies that use hyperbaric exposures for neurodevelopmental research in children have used exposures well below 1.75 ATA, typically 1.2 to 1.4 ATA, often with 100% O_2_. These types of studies are often referred to as “HBOT” but are more accurately described as “mild hyperbaric oxygen therapy” or “mild hyperbaric oxygen”. These same issues of ambiguous terminology are a problem for the evidence synthesis in this manuscript. These studies are thus not equivalent to the typical medical HBOT studies and should not be grouped together in a universal classification system.

In pediatric neurodevelopmental research, various studies have been conducted at sub-1.5 ATA exposures in children with autism. Chungpaibulpatana et al. [21] conducted a pilot study in Thai children with autism. The children were treated with 1.3 atm of hyperbaric oxygen for 10 sessions. The results showed that all five children in the study had improved behaviors. Rossignol et al. [22] conducted a multicenter randomized controlled trial involving 62 children with autism. The children were treated with 1.3 atm of 24% oxygen for 40 sessions (total of 80 hyperbaric exposures). The children were randomized to either mild hyperbaric oxygen therapy or room air control. The results showed no significant differences between the two groups. Jepson et al. [23] conducted a study on 16 children with autism spectrum disorders. The children were treated with 1.3 ATA of 24% oxygen for 40 sessions (total of 80 exposures). The children received behavioral assessments prior to the first exposure and following the last exposure. The results showed no significant improvements in behavior among the children.

A similar problem exists in the cerebral palsy literature, where most controlled trials have been conducted at pressures of 1.3 ATA or higher using ambient air exposures. The Lancet trial [24] used 100% oxygen at 1.75 ATA to treat cerebral palsy, while the control group received slightly pressurized air at 1.3 ATA. In addition, there have been studies of cerebral palsy where children with cerebral palsy have been exposed to slightly pressurized air at 1.3 ATA as treatment and compared to children with cerebral palsy who were exposed to ambient air at 1.0 ATA as controls. In other studies, children with cerebral palsy have been exposed to hyperbaric oxygen at 100% at 1.75 ATA as a treatment and compared to children with cerebral palsy who were exposed to ambient air at 1.3 ATA as controls. Hardy et al. [25] reported a neuropsychological study of children with cerebral palsy in a companion article to the Lancet trial. In this study, children with cerebral palsy were exposed to either hyperbaric oxygen at 100% at 1.75 ATA or ambient air at 1.3 ATA, and Hardy et al. [25] reported significant improvements in all measures of neuropsychological functioning in both groups. Therefore, slightly pressurized air at 1.3 ATA cannot be used as a true control or placebo group in studies of HBOT in cerebral palsy. In addition, the term HBOT must not be used to refer to exposures at pressures of 1.2 to 1.3 ATA of ambient air because these exposures are not equivalent to medical HBOT and have been shown to have positive effects in their own right.

Regarding mild traumatic brain injury (mTBI) and persistent post-concussion symptoms (PSPS), several studies have employed sub-1.5 ATA exposures as both treatment and sham. Wolf and colleagues [26] used 1.3 ATA room air as a sham exposure to 2.4 ATA oxygen in a small pilot study of the effects of hyperoxia after mild traumatic brain injury. The studies conducted by Miller et al. [27] and those involving service members with PSPS are part of a larger controversy regarding whether low-pressure exposures are “biologically active” and therefore not true shams. Weaver and colleagues [28] completed a randomized clinical trial comparing 1.5 ATA oxygen vs. 1.2 ATA room air exposure in individuals with PSPS. A systematic review of hyperbaric oxygen therapy was published by Harch in 2022, in which he included positive and negative studies that employed 1.2 ATA air and 1.3 ATA air exposures as sham in post-concussion patients [29]. In mild hyperbaric oxygen therapy studies employing the sub-1.5 ATA exposures as both treatment and control (sham) exposures, Hu and colleagues [30] criticized the use of 1.2 or 1.3 ATA of room air as an effective sham. However, Figueroa et al. [31] pointed out that such pressurized air exposures (1.2 to 1.3 ATA) are “biologically active” and therefore not a true placebo or control (sham) exposure. As such, sub-1.5 ATA exposures are already recognized in the published literature as biologically significant. Thus, they are treated as having a mechanistic interest rather than as having no effect.

In addition to neurological outcomes, many studies in exercise and human performance have investigated mHBOT protocols. One such study by Takemura and colleagues [32] examined blood pressure changes following exposure to 1.3 ATA of approximately 30% oxygen in healthy humans. The same research group found that 1.3 ATA for 10 min at 31% oxygen following high-intensity exercise could promote mood recovery. Qu and associates [33] investigated recovery from exercise-induced muscle fatigue in young athletic males following 1.25 ATA exposure with 26–28% oxygen. Hu and co-workers [34] reported an acute crossover study in which 12 young healthy males received 60 min of 1.3 ATA with 100% oxygen. The effects of 1.3 ATA with 35% oxygen during exercise testing were also reported by Hisamoto and associates [35], while Nisa et al. [36] used 1.4 ATA with 35–40% oxygen in a crossover study, reporting increased parasympathetic activity and an increased number of circulating natural killer cells. These studies comprise an active research category that should not be confused with 2.0–3.0 ATA medical HBOT.

Preclinical and translational studies using relatively low pressure and/or %O_2_ follow a parallel course. In a 12-week study at 1.25 ATA/36%O_2_, diabetic mice developed cataracts significantly later than their non-diabetic counterparts [37]. Studies in humans using 1.25 ATA/32%O_2_ showed improvement in pigmentation and senile white spots induced by UVB exposure [38]. While Suzuki et al. [39] used 1.3 ATA/20.9% O_2_ to assess the effect of mHBOT on the ability of mice to perform endurance and interval exercise and to determine the optimal exposure time, other studies have assessed the hemodynamic effects of 1.3 ATA in diabetic rats [40]. Such preclinical and translational studies are of mechanistic interest but are not part of the main body of literature describing standard HBOT at higher pressures and %O_2_.

The problem of classification is also illustrated by several recent studies on the treatment of wounds using hyperbaric oxygen. Sack et al. [41] compared transcutaneous oxygen measurements in patients with chronic ulcers treated with 100% oxygen at 1.4 ATA and 2.0 ATA over multiple sessions. They found that the TcPO2 values were very low at 1.4 ATA (mean values ranging from 2.5 mmHg to 9.7 mmHg across patients) and much higher at 2.0 ATA (mean values ranging from 19.3 mmHg to 45.2 mmHg across patients). In addition, Sack and colleagues noted that the low-pressure soft-sided chambers used in some clinics to treat patients on an outpatient basis operate at a pressure of about 1.3 ATA and yield an oxygen partial pressure of about 80 mmHg, which is well below that of hard-sided HBOT chambers. This study would be particularly useful for highlighting the problems of sub-1.5 ATA exposures and equivalence claims in the commercial marketplace.

This large body of evidence represents an area of study examining a distinct set of interventions administered at exposures of less than 1.5 ATA. Rather than further classifying these interventions into subclasses and trying to determine equivalence across the wide range of conditions studied, it would be more beneficial to study these conditions within the large body of existing data as interventions in their own right. These would include an investigation into the potential of mHBOT in children with autism and children with cerebral palsy, as well as the effects of mHBOT in individuals with mild traumatic brain injury and persistent post-concussion-like symptoms, alongside other effects of mild hyperbaric oxygen treatment in various other conditions. Furthermore, sub-1.5 ATA exposures are currently being studied in exercise and performance, as well as regarding immune and metabolic functions in animal models and in skin and other tissues. As such, these studies using sub-1.5 ATA exposures are referred to using various terms. Most of the published studies involving such sub-1.5 ATA exposures have been framed as “HBOT”, and, even within individual studies, certain sessions have been referred to as “mild”. In general, the studies that utilize such sub-1.5 ATA exposures are referred to in the literature as hyperbaric oxygen therapy. The purpose of this review is to define these types of treatments delivered via sub-1.5 ATA exposures, using sub-100% O_2_ gas, within a context to establish a standardized classification so that future reviews, as well as a corresponding increase in the validity of the resulting meta-analyses, may assist in accurately determining effects reported for these studies.

Notably, even though most of the studies described here did not find a significant improvement for most of the measured endpoints, there were positive findings for mHBOT exposures at atmospheric pressure in several instances: exercise recovery, autonomic function, inflammatory biomarkers, immune cell function, and a number of neurobehavioral and neurological endpoints. Thus, there are clearly some biological or clinical effects from mHBOT exposures, but these are not equivalent to the effects of hyperbaric exposures and most likely represent pressure at lower-than-hyperbaric levels that could have clinical relevance and would be worthy of additional scientific investigation. Importantly, the existence of such mHBOT effects at subhyperbaric pressures does not mean that the studies here support the use of mHBOT as a substitute for HBOT. The effects of pressure at subhyperbaric levels likely have their own “dose–response” curve that would occupy a different position on the continuum of pressure-dependent biologic and clinical effects than with HBOT; thus, they would require independent scientific study.

Collectively, the studies shown in Table 2 demonstrate that sub-1.5 ATA hyperbaric exposures have been investigated across diverse populations and indications, including neurological conditions, exercise recovery, immune function, metabolism, dermatology, and animal models. The terminology used to describe these interventions is inconsistent—some studies label the exposures as hyperbaric oxygen therapy (HBOT), while others use the term mild hyperbaric oxygen therapy (mHBOT). Key findings indicate that low-pressure exposures are not physiologically inert; they can produce measurable biological and clinical effects. However, the distinctions in oxygen delivery and pressure underscore the importance of classifying sub-1.5 ATA exposures separately from conventional medical HBOT protocols to ensure clarity in evidence synthesis and clinical claims.

### 7.1. Evidence Supporting Biological Activity at Sub-1.5 ATA Exposures

Although several constraints exist for studies that have used sub-1.5 ATA exposures, the findings suggest that mHBOT is a biologically active intervention. Studies found that out-of-shape patients who received mHBOT after exercise showed enhanced recovery from fatigue, as well as improved autonomic balance, mood, and ability to perform exercise or daily activities. Additionally, in healthy individuals, studies have found that mHBOT affects blood pressure regulation, natural killer cell activity, and levels of inflammatory markers and cytokines following a single hyperbaric exposure. Children and adults with various neurological disorders (autism spectrum disorder, cerebral palsy, and various complaints of persistent post-concussion-like symptoms) have also found various positive effects following exposure to air at low pressures in hyperbaric chambers.

Sub-1.5 ATA exposures are not physiologically inert, and their potential clinical effects should be investigated. However, the effects of such exposures should not be compared to the established effects of conventional HBOT. The vast majority of studies to date that have found positive effects of exposure to sub-1.5 ATA environments are small, uncontrolled studies that vary across parameters (dose, frequency, etc.) and thus should be interpreted in isolation and not be conflated with the existing body of literature on HBOT.

### 7.2. Strengths and Limitations of the Sub-1.5 ATA Literature

A word of caution is warranted when reviewing studies conducted at sub-1.5 ATA, as these studies have many limitations. Small patient numbers, varied patient populations, different oxygen concentrations, and non-uniform treatment protocols and endpoints are just a few of the limitations. Most of the studies published on this topic are phase 0 studies. This means that they are exploratory, feasibility, or pilot-type studies attempting to determine whether a particular end-point can be changed with the study of hyperbaric oxygen. These types of studies are typically underpowered and therefore unable to establish the clinical significance of the noted changes.

Sub-1.5 ATA exposures have been reported in the literature using various methods, including differences in chamber pressure, oxygen concentrations, treatment durations, and comparison therapies. The substantial methodological variation among studies precludes an adequate evaluation of their effects. The majority of studies on exposures to sub-1.5 ATA found effects of hyperbaric oxygen on biological systems. The key issue now is to determine the magnitude and the extent to which these effects are reproducible and clinically relevant.

## 8. Studies Directly Comparing Sub-1.5 ATA and ≥1.5 ATA Exposures

A small but important subset of the hyperbaric literature directly compares exposures delivered at pressures below 1.5 ATA with exposures delivered at 1.5 ATA or higher. These studies are particularly relevant to the present review because they test, within the same protocol, whether lower-pressure exposures behave as inert controls, partial-dose equivalents, or distinct interventions. The available comparative literature suggests that sub-1.5 ATA exposures are often neither physiologically nor clinically inert, and they do not behave as interchangeable equivalents of standard medical-grade HBOT.

The earliest and most widely cited comparative trial is, again, the Collet et al. [24] multicenter randomized study in children with cerebral palsy. In that trial, the active arm received 100% oxygen at 1.75 ATA, while the comparison arm received air at 1.3 ATA. Both groups improved over the study period, but there was no significant between-group difference in terms of gross motor or secondary outcomes. The design is central to the classification question because it directly compared a sub-1.5 ATA exposure with a >1.5 ATA exposure and showed that the lower-pressure exposure could not be assumed to be a neutral placebo.

A related neuropsychological analysis by Hardy et al. used the same cerebral palsy cohort and again compared 1.75 ATA oxygen with 1.3 ATA air [25]. Children in both groups showed improvement in selected neuropsychological measures, but no statistically meaningful superiority of the higher-pressure intervention was demonstrated. This reinforced the possibility that the 1.3 ATA condition was an active exposure rather than a true inert sham.

The largest cluster of direct comparisons comes from the persistent post-concussion syndrome/mild traumatic brain injury literature. In the Air Force trial by Wolf et al. [26], participants were randomized to either 2.4 ATA hyperbaric oxygen or 1.3 ATA room air sham for 30 sessions. Both groups improved over time, but there was no significant advantage for 2.4 ATA HBOT on the principal symptom outcomes. This study is repeatedly cited in the debate over whether 1.3 ATA air should be regarded as a sham, a mild hyperbaric intervention, or a distinct treatment category.

In the multicenter military study by Miller et al., participants were assigned to 1.5 ATA HBOT, a 1.2 ATA room-air sham, or a no-chamber intervention [27]. Both chamber groups improved compared with the no-intervention group, but HBOT at 1.5 ATA did not differ significantly from the 1.2 ATA chamber condition on the primary symptom outcomes. This is one of the clearest direct examples showing that a sub-1.5 ATA exposure can exert measurable effects yet still fail to establish equivalence with standard HBOT.

The later BIMA publications by Weaver et al. [28] used a similar head-to-head design, comparing 1.5 ATA, >99% oxygen with 1.2 ATA room air over 40 sessions. The protocol study established the pressure comparison explicitly, and the subsequent Phase II outcomes study reported improvement at 13 weeks in some symptom domains for the 1.5 ATA oxygen group relative to sham, particularly in participants with PTSD, although effects did not persist uniformly over longer follow-up. These studies are important because they represent one of the few modern randomized programs designed specifically around the distinction between sub-1.5 ATA chamber exposure and ≥1.5 ATA HBOT.

A secondary analysis of the same trial by Churchill et al. [42] also compared 1.5 ATA oxygen with 1.2 ATA air for reaction-time outcomes and found no significant between-group differences over time. As with the parent trial, the study underscores that sub-1.5 ATA exposures can complicate interpretation when used as “sham” comparators because they may not be biologically neutral.

Direct pressure comparison research also appears in the context of wound physiology. A comparative study of chronic wound patients measured tissue oxygenation at 1.4 ATA versus 2.0 ATA, showing that oxygen delivery at 1.4 ATA was markedly lower than at 2.0 ATA [43]. Although this is a physiological rather than symptom-based trial, it is highly relevant mechanistically because it demonstrates that exposures just below or at the definitional threshold are not equivalent to conventional treatment pressures used in wound-care HBOT protocols. This type of comparison strongly supports the separate classification of sub-1.5 ATA and standard therapeutic HBOT exposures.

Systematic reviews and editorials in the post-concussion literature have synthesized these comparative trials and explicitly highlighted the methodological problem created by using 1.2 or 1.3 ATA air as a sham against 1.5 to 2.4 ATA oxygen. Reviews by Harch [29] and Bennett [44] and commentary by Hu et al. [30] all note that the interpretation of the study hinges on whether the lower-pressure arm is considered inert, mildly active, or a distinct treatment exposure. That debate itself supports the present manuscript’s argument: once sub-1.5 ATA exposures are acknowledged to have independent physiological relevance, they should not be merged conceptually or terminologically with medical-grade HBOT.

We should also think about these comparison studies differently. Some trials compared lower-pressure treatments with higher-pressure hyperbaric oxygen therapy, and both groups improved, though not in statistically significant ways. One reason for this could be that the lower-pressure treatment produced a therapeutic benefit, so it was not truly a sham treatment. Another possibility is that the difference in pressure or oxygen was not the main reason people improved. Non-specific effects associated with trial participation, expectations, rehabilitation, spontaneous recovery, regression toward the mean, or other uncontrolled variables may also have contributed substantially to outcomes. The lack of superiority of the higher-pressure treatment does not necessarily mean that lower-pressure treatments work the same way or are just as effective as standard hyperbaric oxygen therapy.

We should be careful when considering the effects of small increases in pressure, e.g., 1.2–1.3 ATA. Even though these changes have been linked to physical changes in experiments, it is still unclear how significant an impact they have and whether they are important for treating patients. For instance, if small changes in air pressure could greatly improve recovery from brain injuries, we would expect to see large differences in recovery across altitudes. However, this has not been shown in medical studies. Nevertheless, pressures below 1.5 ATA might not be completely inert, and further research is needed to determine the full breadth and potential of mHBOT in clinical practice.

## 9. Traditional Hyperbaric Oxygen Therapy (HBOT) at Pressures ≥2.0 ATA: Evidence Base Underlying Guidelines and Payer Adoption

The modern clinical framework for hyperbaric oxygen therapy (HBOT) is rooted in a body of literature utilizing full-body chamber exposures at pressures typically ranging from 2.0 to 3.0 atmospheres absolute (ATA) with near-100% oxygen delivery. This pressure range defines what is considered “true” HBOT in the context of guideline development and insurance coverage policy. The Undersea and Hyperbaric Medical Society (UHMS) has been the primary authority synthesizing this literature into formal indications, and its recommendations have been widely adopted by payers, including the Centers for Medicare & Medicaid Services (CMS), which restricts reimbursement to a defined set of conditions supported by this high-pressure evidence base [2,45].

Importantly, the studies that informed these recommendations were conducted using pressures ≥2.0 ATA, and often higher (2.4–3.0 ATA), reflecting the physiologic threshold required to achieve substantial increases in dissolved plasma oxygen, enhanced diffusion gradients, and downstream cellular effects such as angiogenesis, leukocyte activation, and toxin inhibition [3]. This distinction is critical when interpreting the literature that insurers have historically used to define “medically necessary” HBOT.

One of the most influential randomized controlled trials in HBOT literature is the study by Weaver et al., published in the New England Journal of Medicine (2002) [46]. This trial evaluated symptomatic acute carbon monoxide (CO) poisoning and utilized a treatment protocol beginning at 3.0 ATA. The study demonstrated that three HBOT sessions within 24 h significantly reduced the incidence of delayed neurologic sequelae at both six weeks and twelve months compared to normobaric oxygen.

Chronic diabetic wounds represent another major category where high-pressure HBOT literature has driven payer acceptance. Multiple studies using treatment pressures of approximately 2.4–2.5 ATA have demonstrated improved healing outcomes and reduced amputation rates.

The randomized study conducted by Faglia et al. showed that adjunctive HBOT significantly reduced the risk of major amputation in patients with severe diabetic foot ulcers [47]. Similarly, Kessler et al. reported accelerated healing rates in patients treated at 2.5 ATA compared to standard care alone [48]. The Hyperbaric Oxygen Therapy in Diabetics with Chronic Foot Ulcers (HODFU) trial further reinforced these findings, demonstrating improved complete healing rates at one year [43].

The foundational research supporting HBOT in delayed radiation injury comes from Marx. His 1985 randomized controlled trial demonstrated that prophylactic HBOT significantly reduced the incidence of mandibular osteoradionecrosis in irradiated patients undergoing dental extraction compared to antibiotics alone [49].

Future research could further investigate the use of HBOT for various musculoskeletal disorders. Recently, a systematic review on the use of HBOT for musculoskeletal pain of chronic or sports-related origin or of inflammatory origin was published by Martín Pérez et al. [50]. The review includes 32 studies. Although evidence from the included studies supports the use of HBOT in these cases, study quality varies widely, and more research is warranted, particularly high-quality randomized controlled trials. All studies included in the review were conducted at pressures typical of conventional HBOT, not at the sub-1.5 ATA pressures typical of mHBOT. Therefore, although the evidence base for the use of HBOT in various situations is large, the evidence base for mHBOT is relatively small.

This research provided both clinical outcomes data and a biologically plausible mechanism: HBOT at pressures above 2.0 ATA enhances angiogenesis, fibroblast proliferation, collagen synthesis, and capillary density in hypoxic irradiated tissues [1]. These effects directly address the pathophysiology of radiation-induced tissue damage, which is characterized by hypovascularity, hypocellularity, and chronic hypoxia.

Across all major approved indications, a consistent pattern emerges: the evidence base that informed guideline development and payer coverage decisions is overwhelmingly derived from studies utilizing pressures ≥2.0 ATA. These pressures are necessary to achieve the physiologic effects that define HBOT’s therapeutic value—namely, substantial increases in dissolved oxygen content, enhanced tissue oxygen gradients, and downstream cellular and vascular responses [4].

## 10. Limitations of the Current Evidence Base

There are limitations to the mHBOT research conducted to date, as well as to the current review. Most research to date has focused on the effects of mHBOT at sub-1.5 ATA pressures, with relatively small sample sizes. These studies lack the statistical power to identify all of the potential effects of mHBOT. There is considerable variability among studies for several key parameters, including chamber pressure, oxygen concentration, treatment duration, treatment frequency, and the number of treatments administered. In terms of outcome measures, some studies report various physiological and/or mechanistic endpoints, whereas others report more clinically relevant endpoints. There is also inconsistent use of terminology throughout the literature reviewed here, which introduces potential errors into the synthesis of evidence and the risk of mHBOT interventions being misclassified. The current review is a narrative review and is thus subject to elements of selection bias. The review was conducted using a well-structured search strategy; however, the review is not a formally conducted systematic review or meta-analysis. As such, additional, larger randomized controlled trials will likely be needed to more fully establish the clinical role of mHBOT. In addition, more consistent use of nomenclature across studies, as well as direct comparisons of effects across different pressure and oxygen-dose ranges, will be necessary.

## 11. Conclusions

The cumulative literature base suggests three conclusions. First, head-to-head studies comparing <1.5 ATA with ≥1.5 ATA exist, but the literature is relatively sparse and concentrated in a few indications. Second, sub-1.5 ATA chamber exposures often produce measurable change, which makes them problematic as “placebos.” Third, higher-pressure HBOT and lower-pressure exposures are not interchangeable interventions, either clinically or mechanistically. These comparative studies therefore support the need for a universal classification framework that distinguishes mild hyperbaric exposure from therapeutic HBOT based on both pressure and oxygen dose.

The evidence reviewed in the present study suggests that conventional HBOT and lower-pressure hyperbaric exposures should not automatically be assumed to be clinically or mechanistically equivalent. Although overlapping biological responses may occur across a continuum of pressure and oxygen exposure, important differences exist in oxygen delivery, evidence strength, regulatory status, and demonstrated clinical efficacy. Additional mechanistic and clinical studies are needed to determine the extent to which these interventions share common pathways and whether specific therapeutic thresholds exist for particular medical conditions.

## Figures and Tables

**Table 1:** Proposed standardized classification of hyperbaric therapies based on pressure and oxygen delivery.

Category	Ambient Pressure (ATA)	Oxygen Delivery	Oxygen Partial Pressure	Typical Setting	Clinical Status	Terminology Recommendation
Normobaric Oxygen	1.0 ATA	21–100% O_2_	≤760 mmHg	Clinical/Home	Established	Normobaric Oxygen Therapy
Mild Hyperbaric Therapy (mHBOT)	1.1–1.49 ATA	Ambient air or low-flow O_2_	~250–400 mmHg	Wellness/outpatient	Not FDA cleared	Mild Hyperbaric Therapy (mHBOT)
Medical-Grade HBOT	≥1.5 ATA (typically 2.0–3.0 ATA)	Near-100% O_2_	1500–2000+ mmHg	Hospital/medical clinic	FDA cleared indications	Hyperbaric Oxygen Therapy (HBOT)

**Table 2:** A summary of published studies utilizing sub-1.5 ATA hyperbaric exposure, organized by clinical indication and evidence strength.

Clinical Indication/Population	Representative Studies	Study Design	Pressure Range	Principal Findings	Evidence Quality *
Autism Spectrum Disorder	Rossignol et al. [22]; Granpeesheh et al. [23]	Randomized controlled trials; pilot studies	1.3 ATA, 24–28% O_2_	Improvements reported in selected behavioral, communication, and social function measures; findings inconsistent across studies	Moderate
Cerebral Palsy	Collet et al. [24]	Randomized controlled trials	1.3 ATA	Improvements observed in both treatment and comparison groups; difficulty distinguishing treatment effects from non-specific effects	Moderate
Persistent Post-Concussion Syndrome/Mild Traumatic Brain Injury	Miller et al. [27]; Harch et al. [29];	Randomized controlled trials; observational studies	1.2–1.5 ATA	Symptom improvement reported in several studies; many trials demonstrated improvement in both intervention and control groups	Moderate
Neurological Rehabilitation (Mixed Conditions)	Various pilot studies	Pilot trials; observational studies	1.2–1.5 ATA	Improvements in selected neurocognitive and quality-of-life outcomes reported; findings require replication	Low
Exercise Recovery and Athletic Performance	Suzuki et al. [39];	Controlled human studies	1.2–1.3 ATA	Improvements reported in fatigue recovery, autonomic regulation, mood state, and post-exercise recovery metrics	Moderate
Healthy Volunteers	Various physiological studies	Controlled laboratory studies	1.2–1.3 ATA	Demonstrated physiological responses including changes in autonomic function, inflammatory markers, vascular responses, and immune activity	Moderate
Animal Models	Various experimental studies	Preclinical studies	1.2–1.5 ATA	Demonstrated biological activity affecting inflammation, oxidative stress, wound healing, and tissue repair pathways	Low–Moderate
Chronic Wounds/Tissue Repair	Limited pilot studies	Pilot clinical studies	1.2–1.5 ATA	Some evidence of improved tissue oxygenation and healing-related biomarkers; insufficient evidence for definitive conclusions	Low
Immune and Inflammatory Modulation	Various mechanistic studies	Experimental human and animal studies	1.2–1.5 ATA	Changes reported in cytokine activity, natural killer cell function, and inflammatory signaling pathways	Low–Moderate

* Evidence Quality Definitions: High = Multiple adequately powered randomized controlled trials with consistent findings. Moderate = Randomized trials available, but limited by sample size, heterogeneity, or inconsistent findings. Low = Primarily pilot studies, observational studies, mechanistic studies, or limited clinical data.

## Data Availability

No new data were created or analyzed in this study.

## Abbreviations

The following abbreviations are used in this manuscript:

HBOT	Hyperbaric oxygen therapy
mHBOT	Mild hyperbaric oxygen therapy
ATM	Atmospheres
ATA	Absolute atmospheres
